# The incidence of albuminuria after bariatric surgery and usual care in swedish obese subjects (SOS): a prospective controlled intervention trial

**DOI:** 10.1038/ijo.2014.72

**Published:** 2014-06-10

**Authors:** L M S Carlsson, S Romeo, P Jacobson, M A Burza, C Maglio, K Sjöholm, P-A Svensson, B Haraldsson, M Peltonen, L Sjöström

**Affiliations:** 1 Department of Molecular and Clinical Medicine, Institute of Medicine, The Sahlgrenska Academy at University of Gothenburg, Gothenburg, Sweden; 2 Department of Chronic Disease Prevention, National Institute for Health and Welfare, Helsinki, Finland

**Keywords:** Bariatric Surgery, Albuminuria, renal function

## Abstract

**Background:**

Obesity is associated with increased risk of chronic kidney disease and albuminuria is a predictor of renal impairment. Bariatric surgery reduces body weight in obese subjects, but it is not known whether surgery can prevent development of albuminuria. This study aims to determine the long-term effect of bariatric surgery on the incidence of albuminuria.

**Subjects:**

The Swedish Obese Subjects study is a non-randomized, prospective, controlled study conducted at 25 public surgical departments and 480 primary health care centers in Sweden. Between 1 September 1987 and 31 January 2001, 2010 participants who underwent bariatric surgery and 2037 controls were recruited. Inclusion criteria were age 37–60 years and BMI⩾34 in men and BMI⩾38 in women. In this analysis, we included 1498 patients in the surgery group and 1610 controls without albuminuria at baseline. Patients in the bariatric surgery group underwent banding (18%), vertical banded gastroplasty (69%) or gastric bypass (13%); controls received usual obesity care. Date of analysis was 1 January 2011. Median follow-up was 10 years, and the rates of follow-up were 87%, 74 and 52% at 2, 10 and 15 years, respectively. The main outcome of this report is incidence of albuminuria (defined as urinary albumin excretion >30 mg per 24 h) over up to 15 years.

**Results:**

During the follow-up, albuminuria developed in 246 participants in the control group and in 126 in the bariatric surgery group, corresponding to incidence rates of 20.4 and 9.4 per 1000 person years, respectively (adjusted hazard ratio, 0.37; 95% confidence interval, 0.30–0.47; *P*<0.001). The expected number of surgeries needed to prevent the development of albuminuria in one patient at 10 years was nine.

**Conclusions:**

Bariatric surgery is associated with reduced incidence of albuminuria compared with usual obesity care.

## Introduction

The prevalence of obesity has increased dramatically over recent decades and approximately one-third of the adult population of the United States today are obese.^1^ It is well established that obesity is associated with an increased risk of type 2 diabetes, hypertension and cardiovascular disease,^2, 3, 4, 5^ all of which may be involved in the etiology of chronic kidney disease. In addition, there is increasing evidence that obesity is an independent risk factor for chronic kidney disease.^6,7^

Albuminuria is a well-accepted predictor of chronic kidney disease^8,9^ and the prevalence of albuminuria is increased in obese individuals.^10,11^ One suggested link between obesity and albuminuria is increased intraabdominal pressure caused by increased amounts of visceral adipose tissue, resulting in venous stasis of the kidneys.^12, 13, 14^ Lifestyle changes that reduce body weight are known to reduce albuminuria.^15^ Bariatric surgery is the most effective treatment to achieve and maintain weight loss in severely obese patients,^16^ and previous studies indicate that weight-loss surgery also reduces the prevalence of albuminuria.^17, 18, 19, 20, 21, 22^ In a small study by Iaconelli *et al.*,^17^ seven patients with microalbuminuria at baseline had recovered from albuminuria and 15 patients remained free from albuminuria 10 years after biliopancreatic diversion, whereas the prevalence of albuminuria had increased in the non-surgical control group. However, there are no reports that specifically examine the long-term effect of bariatric surgery on the prevention of albuminuria in obese individuals without albuminuria at baseline.

The SOS study is a non-randomized, prospective, controlled, intervention trial that compares the long-term effects of bariatric surgery and usual care in obese subjects. We have previously reported positive effects of bariatric surgery on overall mortality (the predefined primary endpoint of the SOS study), cardiovascular disease and diabetes (predefined secondary endpoints).^16,23, 24, 25^ Although albuminuria was not a predefined endpoint in SOS, all data were collected prospectively, and we here report the long-term effects of bariatric surgery on albuminuria incidence.

## Subjects and methods

### Study design

The SOS study is a prospective, controlled intervention study conducted at 25 public surgical departments and 480 primary health care centers in Sweden. The design has been previously described.^16,26^ In brief, 2010 participants who underwent bariatric surgery and 2037 controls, were recruited between 1 September 1987 and 31 January 2001. Inclusion criteria were age between 37 and 60 years and body mass index (BMI) of 34 kg m^2^ or more for men and 38 kg m^2^ or more for women. The exclusion criteria were the same in both groups and designed to obtain an operable surgery group.

For the current analysis, SOS study participants were excluded from if they had albuminuria at baseline (*n*=811), if baseline information on diabetes or hypertension status (*n*=12), urine sample (*n*=31) or urinary albumin concentration (*n*=8) were not available or if urine collection time was shorter than 20 h (*n*=77). The current analysis therefore includes 1498 patients who underwent bariatric surgery and 1610 controls (Table 1). Participants in the surgery group underwent nonadjustable or adjustable banding (18%), vertical banded gastroplasty (69%) or gastric bypass (13%). The participants in the control group were given conventional non-surgical treatment for obesity at their primary health care centers. No attempt was made to standardize the non-surgical treatment.

### Data collection and definitions

The baseline examination took place approximately 4 weeks before the date of bariatric surgery for both the surgery patients and the controls. Physical examination was undertaken at the baseline examination and after 0.5, 1, 2, 3, 4, 6, 8, 10 and 15 years. Fasting blood samples and 24 h urine collections were obtained and biochemical assays were performed at the baseline visit and after 2, 10 and 15 years at the Central Laboratory, Sahlgrenska University Hospital, Gothenburg, Sweden (accredited according to European Norm 45001). The 24-h urine collection was performed at home according to detailed instructions. Urinary albumin excretion was calculated based on the 24-h urine collection, according to the formula: (urine albumin concentration (mg l^−1^) × urine volume (l))/urine collection time (min) and expressed as mg per 24 h. Albuminuria was defined as urinary albumin excretion of 30 mg/24 h or more (corresponding to 20 μg/min or more).^27^ Impaired fasting glucose was defined as fasting blood glucose of at least 90 mg dl^−1^ and less than 110 mg dl^−1^ (corresponding to fasting blood glucose of 5.0 mmol l^−1^ or more and less than 6.1 mmol l^−1^). Type 2 diabetes was defined as fasting blood glucose of 110 mg dl^−1^ or more (corresponding to 6.1 mmol l^−1^ or more) and/or therapy with glucose-lowering medications.^28,29^ Hypertension was defined as systolic blood pressure of at least 140 mm Hg or diastolic blood pressure of at least 90 mm Hg or treatment with antihypertensive medication.^30^ The sagittal diameter, measured as the distance between the examination table and a carpenter's level held horizontally across the abdomen at the level of the iliac crest, was used as an index of intraabdominal pressure and visceral adiposity.^12^

The relevant regional ethics review boards in Sweden approved the study protocol and informed consent was obtained from all participants. The study has been registered at ClinicalTrials.gov (NCT01479452).

### Statistical analyses

Mean values, with s.d. and percentages were used to describe the baseline characteristics. Baseline differences between treatment groups were evaluated by using *t*-tests for continuous variables and Fisher's exact text for categorical variables. Participants were followed up either until the diagnosis of albuminuria, or until their last follow-up examination, whichever occurred first. Those without albuminuria during follow-up were thus censored at their last follow-up visit.

Time to albuminuria diagnosis was compared between the bariatric surgery and control groups with Kaplan–Meier estimates of cumulative incidence rates. The log-rank test was used to examine differences in the cumulative incidence. The hazard ratio from a Cox proportional-hazards model with a single covariate for treatment group (surgery or control) was calculated. In addition, hazard ratios were calculated adjusted for age, gender, waist hip ratio, sagittal diameter, diabetes, hypertension, triglycerides and albumin excretion at baseline using a multivariate Cox regression model.

In secondary subgroup analyses, the cumulative incidence of albuminuria was calculated separately in subgroups defined according to baseline parameters. The association between risk factors and the effect of bariatric surgery on the development of albuminuria was tested by including the corresponding interaction term in the Cox proportional hazard regression model. In these calculations, dichotomous variables could have one of the two values (for example, male or female sex). For other traits, the interaction tests were conducted using the original continuous variable, dichotomised based on median baseline values. A total of 18 *post hoc* treatment interaction analyses were performed.

The number needed to treat to prevent the development of albuminuria in one patient at 10 years was calculated in different subgroups as the reciprocal of the absolute risk difference between the bariatric surgery and control groups.

All *P*-values are two-tailed and *P*<0.05 was considered to be statistically significant. In all calculations, the intention-to-treat principle was applied. The Stata statistical package, version 10.1 (StataCorp, College Station, TX, USA), was used.

## Results

### Baseline characteristics, follow-up rates and weight changes during follow-up

Characteristics of the SOS participants included in this report are shown in Table 1. For most of the baseline characteristics, participants in the surgery group were more metabolically deranged than the controls. At baseline, mean BMI was higher in the bariatric surgery group compared with the control group, and the surgery group had a higher proportion of individuals with risk factors for albuminuria (type 2 diabetes, hypertension and smokers) compared with the control group.

The median follow-up time in this analysis was 10 years (interquartile range 2 to 10 years, maximum 15 years). After adjustment for mortality, the rates of follow-up were 87% at 2 years and 74% at 10 years. After adjustment, also for those patients who had not yet been followed for 15 years at the time of analysis, the follow-up rate was 52% at 15 years.

**Figure 1:**
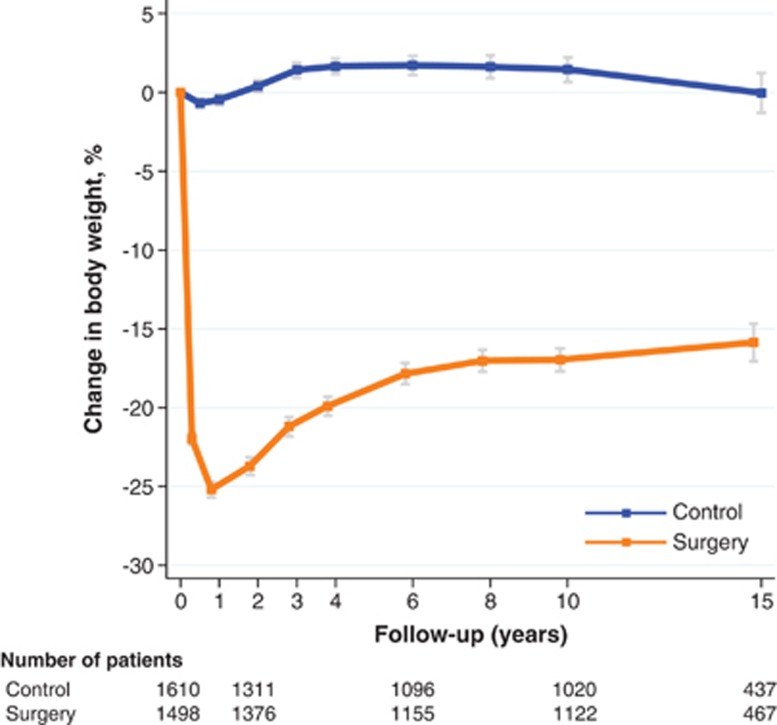
Mean body weight changes (%) and 95% confidence intervals (bars) over 15 years in the control and surgery groups of the SOS study.

No significant weight loss was observed in the control group during 15 years of follow-up (Figure 1). In contrast, the average weight loss in the bariatric surgery group was 25% after 1 year. After partial weight regain, the average weight loss at 15 years was 16% (Figure 1; *P*<0.001 vs controls). At all the time points we examined, the weight loss was greater after gastric bypass than after banding or vertical banded gastroplasty (Supplementary Figure 1).

### Incidence of albuminuria

**Figure 2:**
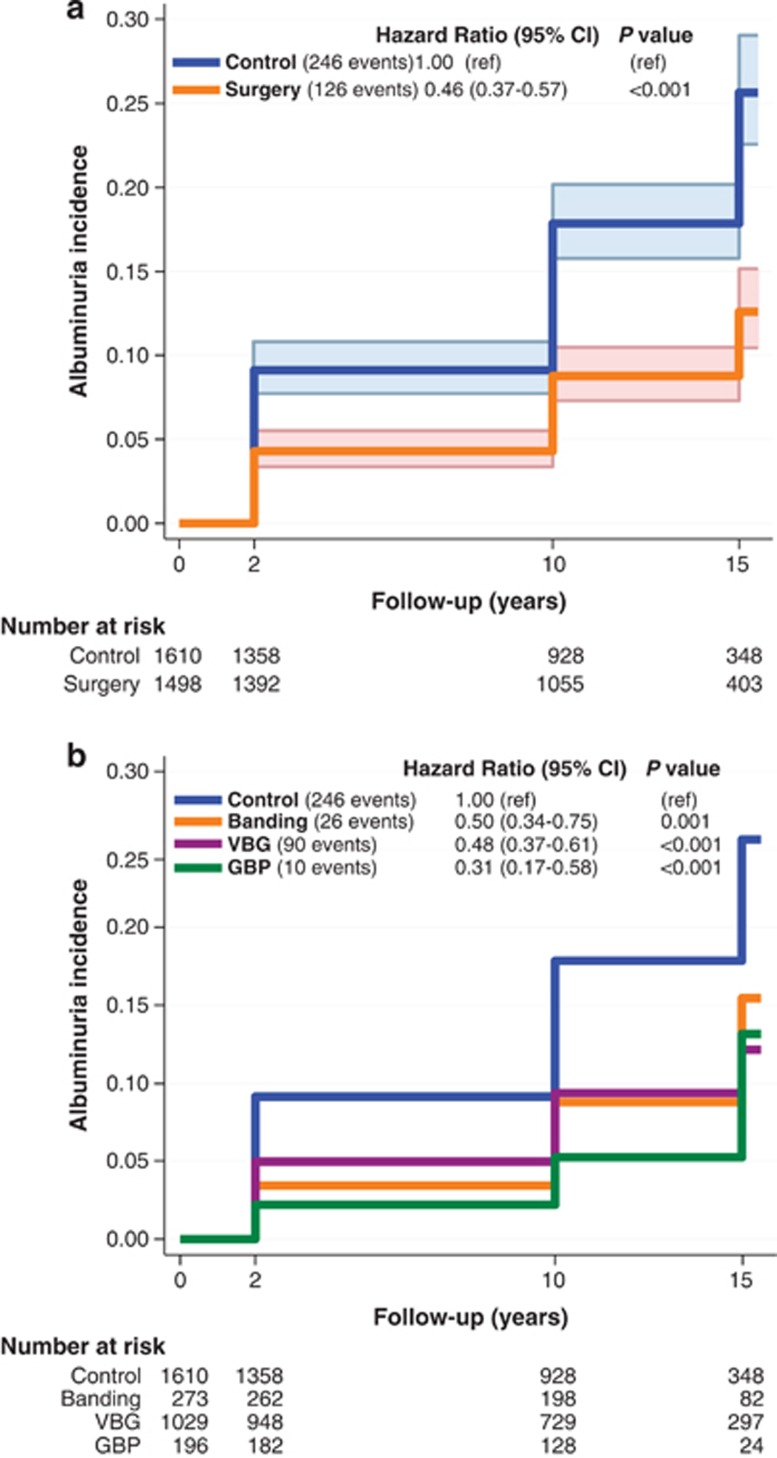
Kaplan–Meier estimates of cumulative incidence of albuminuria in the control group compared with the entire surgery group (**a**) and to the surgery group plotted according to the method of bariatric surgery (**b**). Albuminuria was defined as urinary albumin excretion equal to or above 30 mg per 24 h or more. The unadjusted hazard ratio and the 95% CI are shown in the figure using the control group as reference.

During the follow-up period, albuminuria developed in 246 patients in the control group and 126 patients in the bariatric surgery group (Figure 2a), corresponding to incidence rates of 20.4 cases (95% confidence interva (CI), 18.0 to 23.2) and 9.4 cases (95% CI, 7.9 to 11.2) per 1000 person years, respectively (log-rank *P*<0.001; unadjusted hazard ratio=0.46, 95% CI, 0.37–0.57). After adjustment for confounding baseline factors (age, gender, BMI, sagittal diameter, diabetes, hypertension, triglycerides and urinary albumin excretion), the hazard ratio was 0.37 (Table 2).

### Subgroup analysis

All three types of bariatric surgery were associated with a reduced incidence of albuminuria compared with usual care (*P*⩽0.001; Figure 2b). Within the surgery group, the hazard ratios for albuminuria in the banding and vertical banded gastroplasty groups as compared with the GBP group were 1.62 (95% CI 0.78–3.34, *P*=0.19) and 1.54 (95% CI 0.80–2.94, *P*=0.20), respectively (Figure 2b). The non-significant *P*-values remained after exclusion of patients in the banding and vertical banded gastroplasty groups who were converted to GBP during follow-up. However, it should be noted that the number of events in these analyses were low, affecting the power of the tests.

The incidence of albuminuria in subgroups defined according to baseline parameters and interactions between baseline risk factors and treatment are shown in Table 3. In the control group, many high-risk subgroups (for example, male sex, diabetes, hypertension, high sagittal diameter, high insulin, high glucose, high urine albumin excretion) had a higher incidence of albuminuria than the corresponding low-risk subgroups (Panel A in Table 3).

The association between bariatric surgery and reduced incidence of albuminuria was significant in all subgroups with hazard ratios ranging from 0.35 to 0.55 (Panel B in Table 3). However, the relative treatment effects of bariatric surgery were not significantly different between any of the subgroups (Panel B in Table 3).

The estimated number needed to treat to prevent one case of albuminuria over 10 years in the entire study group was 9 (95% CI 7.1–12.5; Panel C in Table 3). A difference in the number needed to treat between subgroups defined by baseline factors was found when the SOS cohort was subdivided by median baseline urine albumin excretion (9.3 mg per 24 h), serum triglycerides (154 mg dl^−1^), diabetes and sex. In individuals with baseline urine albumin excretion below or equal to the median, the number needed to treat was fourfold higher than in individuals with albumin excretion rates greater than median at baseline (Panel C in Table 3).

## Discussion

In the current report, we examined the long-term effects of bariatric surgery on albumin excretion in patients without albuminuria at study start. Our results show that bariatric surgery is associated with more than 50% lower incidence of albuminuria compared with conventional obesity treatment. Although gastric bypass resulted in greater weight loss, the preventive effect on albuminuria was not different compared with the restrictive procedures but this should be interpreted with caution because the SOS study was not designed to detect such differences. In the whole cohort, surgical treatment of nine patients was needed to prevent the development of albuminuria in one patient at 10 years. However, in some high-risk subgroups (men, diabetics and participants with high triglyceride levels or high urinary albumin excretion at study start), the number of surgeries needed to prevent the development of albuminuria in one patient was lower than in the corresponding low-risk groups. In these high-risk groups, one case of albuminuria was prevented for every four to six surgeries. It should also be noted that, although all participants in this study were free from albuminuria (baseline urinary albumin excretion less than 30 mg per 24 h), baseline urine albumin excretion above the median (9.3 mg per 24 h) was associated with increased progression to albuminuria.

The mechanisms behind the beneficial effects of bariatric surgery on albuminuria are largely unknown. Obesity *per se* is an independent risk factor for renal disease^6,7^ and is associated with hemodynamic, structural and functional changes of the kidney as well as increased albuminuria.^6,31^ In addition, metabolic syndrome, diabetes and hypertension, well established as major causes of chronic kidney disease,^32^ are closely associated with obesity.^2, 3, 4^ The effect of bariatric surgery on albuminuria incidence may be mediated by the improvement of several risk factors for this condition (for example, diabetes, hypertension and metabolic syndrome). It has also been shown that western style diet with high content of sodium, fat and protein from red meat increases the risk of chronic kidney disease.^33^ It is therefore possible that the dramatic reduction in food intake after bariatric surgery protects against kidney damage and contributes to the reduced incidence of albuminuria in our study.

Interestingly, although obesity is associated with increased risk for albuminuria,^10,11^ the incidence of albuminuria in the control group during the 15-year follow-up was almost identical in subjects with baseline BMI above and below the median in the SOS cohort. In contrast, albuminuria incidence in the control group was higher in individuals with high sagittal diameter, a marker for visceral fat and intraabdominal pressure.^12^ In addition, higher baseline fasting insulin, glucose and triglycerides and lower HDL cholesterol were associated with increased albuminuria incidence in the control group, indicating that metabolic parameters rather than BMI are predictors of an increased incidence of albuminuria among obese individuals. Obesity is characterized by a varying degree of resistance to the physiological effect of insulin. Recent observations suggest that insulin signaling in podocytes, cells having a pivotal role in kidney filtration, contributes to albuminuria.^34, 35, 36^ This could potentially explain why albuminuria incidence is associated with metabolic features rather than with BMI *per se* in the control group in the present study. Our results also show that the effect of bariatric surgery on albuminuria incidence was similar for subgroups with high and low BMI at baseline. This is in line with our earlier observations that baseline BMI does not predict the treatment benefit of bariatric surgery in obese subjects.^24,25,37^

A limitation of this study is that intervention in the SOS study was not randomized owing to ethical considerations related to the high postoperative mortality associated with bariatric surgery in the 1980s.^38^ In addition, there were baseline differences between the surgery and control groups in the subgroup of the original SOS cohort used for the current study. However, the risk factors were worse in the surgery group compared with the control group, and, if anything, our data may therefore underestimate the effects of surgery compared with conventional management. Another limitation is that the SOS study was not primarily designed to assess the effect of bariatric surgery on albuminuria incidence; nonetheless all the analyzed variables were collected prospectively. Further longitudinal randomized-controlled trials are therefore warranted to confirm our results. A strength of the study is that albuminuria measurement was based on a 24 h urine collection. This method is a more reliable way to assess albuminuria in morbidly obese individuals compared with other parameters (for example, albumin-to-creatinine ratio).^39^ As the collection was performed by the patients, there is a risk of over- or under collection of 24 h urine; however, this is likely to affect both treatment groups.

In conclusion, our results show for the first time that bariatric surgery is associated with reduced long-term incidence of albuminuria in obese individuals. Baseline BMI did not influence the incidence of albuminuria in the conventionally treated control group or the treatment benefit in the surgery group.

## Figures and Tables

**Table 1:** Baseline characteristics of study participants

	*Control*	*Surgery*	P*-value*
*N*	1610	1498	
Age, years	48.7±6.2	47.2±6.0	<0.001
Male gender, %	25.5	23.7	0.237
Diabetes, %	9.8	12.5	0.016
Impaired fasting glucose, %	14.3	15.2	0.490
Hypertension, %	60.4	75.2	<0.001
Smoking, %	20.3	24.6	0.004
BMI, kg m^2^	40.0±4.6	42.3±4.4	<0.001
Sagittal diameter, cm	27.1±3.6	28.5±3.5	<0.001
Weight, kg	113.5±15.8	119.3±15.8	<0.001
Systolic blood pressure, mm Hg	136.3±17.3	142.8±17.5	<0.001
Diastolic blood pressure, mm Hg	84.1±10.1	88.6±10.5	<0.001
Blood glucose, mg dl^−1^	85.2±26.6	89.0±31.0	<0.001
Insulin, mU l^−1^	17.1±10.8	20.3±13.0	<0.001
Triglycerides, mg dl^−1^	166.3±97.5	190.8±118.3	<0.001
Total cholesterol, mg dl^−1^	215.1±39.9	226.5±44.1	<0.001
HDL cholesterol, mg dl^−1^	52.7±12.6	53.2±12.4	0.259
Creatinine, mg dl^−1^	0.78±0.10	0.78±0.10	0.538
UAE, mg per 24 h	10.8±6.3	11.4±6.5	0.009

Abbreviations:

BMI, body mass index; HDL, high-density lipoprotein; N, number; UAE, urinary albumin excretion.

Data are expressed as mean±s.d. or proportions. *P*-values were calculated by using *t*-test for continuous variables and Fisher's exact test for categorical variables. To convert the values for glucose to millimoles per liter, multiply by 0.05551. To convert the values for insulin to picomoles per liter, multiply by 6.945. To convert the values for cholesterol to millimoles per liter, multiply by 0.02586. To convert the values for creatinine to micromoles per liter, multiply by 88.4. To convert the values for triglycerides to millimoles per liter, multiply by 0.01129.

**Table 2:** Adjusted hazard ratios for the incidence of albuminuria

	*Adjusted hazard ratios*			
	*HR*	*95% CI*	z	P
Surgery, yes/no	0.37	0.30–0.47	−8.4	<0.001
Sex, men/women	1.91	1.47–2.47	4.9	<0.001
Age, per 6.2 years	0.98	0.88–1.09	−0.4	0.594
BMI, per 4.6 kg m^2^	1.16	1.003–1.35	2.0	0.045
Sagittal diameter, per 3.6 cm	0.92	0.79–1.06	−1.1	0.256
Diabetes, yes/no	1.18	1.08–1.28	3.7	<0.001
Hypertension, yes/no	1.21	0.95–1.55	1.6	0.119
Triglycerides, per 108.7 mg dl^−1^	1.09	1.02–1.17	2.5	0.011
U-albumin excretion, per 6.4 mg per 24 h	1.78	1.63–1.94	12.8	<0.001

Abbreviation: U-albumin, urine albumin.

The adjusted hazard ratios were calculated with the use of a multivariable Cox proportional hazard regression model. The hazard ratios for continuous variables are expressed per 1 s.d. difference at baseline in the study population.

**Table 3:** The incidence of albuminuria, risk factor-treatment interaction analyses and numbers needed to treat

*Variable subgrouping at baseline (*N *surgery/*N *control)*	*A. Incidence per 1000 person years (95% CI)*		*B. Relative treatment effects*				*C*
	*Surgery*	*Control*	*HR*	*95% CI for HR*	P*-value for HR*	P*-value for interaction*^a^	*NNT*
Total (*N* 1498/1610)	9.4 (7.9–11.2)	20.4 (18.0–23.2)	0.46	0.37–0.57	<0.001	-	9
*Gender*						0.271	
Male (*N* 355/411)	14.4 (10.8–19.1)	35.2 (29.5–42.4)	0.40	0.28–0.56	<0.001		5^b^
Female (*N* 1143/1199)	7.8 (6.2–9.7)	15.3 (12.9–18.0)	0.52	0.39–0.68	<0.001		13
*P* (log-rank test)		<0.001					
							
*Age, years*						0.926	
⩽47.9 (N 828/727)	9.9 (7.9–12.5)	21.2 (17.7–25.5)	0.47	0.35–0.63	<0.001		9
>47.9 (N 670/883)	8.7 (6.7–11.4)	19.8 (16.7–23.5)	0.44	0.32–0.61	<0.001		9
*P* (log-rank test)		0.584					
							
*Smoking, No/yes*						0.930	
No (*N* 1129/1279)	8.6 (7.0–10.6)	18.7 (16.2–21.6)	0.46	0.36–0.59	<0.001		10
Yes (*N* 368/325)	12.2 (8.8–17.0)	28.4 (21.9–36.9)	0.44	0.29–0.66	<0.001		6
*P* (log-rank test)		0.012					
							
*BMI, kg* *m*^*2*^						0.340	
⩽40.6 (*N* 581/973)	8.6 (6.5–11.5)	20.1 (17.2–23.6)	0.43	0.31–0.60	<0.001		9
>40.6 (*N* 917/637)	9.9 (7.9–12.3)	21.0 (17.1–25.7)	0.48	0.35–0.64	<0.001		9
*P* (log-rank test)		0.874					
							
*Sagittal diameter, cm*						0.760	
⩽28 (*N* 800/1086)	7.7 (6.5–11.5)	17.9 (15.3–21.0)	0.43	0.32–0.59	<0.001		10
>28 (*N* 692/524)	11.3 (8.9–14.3)	26.3 (21.5–32.2)	0.43	0.31–0.58	<0.001		7
*P* (log-rank test)		0.004					
							
*Energy intake, kcal*						0.850	
⩽2494.6 (*N* 672/882)	7.0 (5.2–9.5)	19.1 (16.0–22.8)	0.37	0.26–0.53	<0.001		8
>2494.6 (*N* 826/727)	11.4 (9.2–14.2)	22.0 (18.4–26.2)	0.52	0.39–0.68	<0.001		10
*P* (log-rank test)		0.206					
							
*Systolic blood pressure, mm Hg*						0.611	
⩽140 (*N* 816/1090)	8.6 (6.7–11.1)	18.6 (15.9–21.8)	0.46	0.34–0.62	<0.001		10
>140 (*N* 679/517)	10.1 (7.9–13.0)	24.8 (20.2–30.5)	0.41	0.30–0.57	<0.001		7
*P* (log-rank test)		0.033					
							
*Diastolic blood pressure, mm Hg*						0.495	
⩽85 (*N* 657/954)	7.6 (5.6–10.2)	16.9 (14.1–20.1)	0.45	0.31–0.63	<0.001		11
>85 (*N* 838/651)	10.6 (8.5–13.1)	26.0 (21.8–31.0)	0.41	0.31–0.54	<0.001		7
*P* (log-rank test)		<0.001					
							
*Hypertension, No/yes*						0.867	
No (*N* 372/637)	7.0 (4.6–10.7)	14.9 (11.8–18.7)	0.47	0.29–0.75	0.002		13
Yes (*N* 1126/973)	10.1 (8.3–12.2)	24.4 (21.0–28.1)	0.42	0.33–0.53	<0.001		7
*P* (log-rank test)		<0.001					
							
*Blood glucose, mg* *dl*^−1^						0.593	
⩽79.4 (*N* 701/864)	7.8 (5.9–10.3)	16.9 (14.1–20.3)	0.46	0.33–0.64	<0.001		11
>79.4 (*N* 796/746)	10.9 (8.7–13.6)	24.9 (21.0–29.5)	0.44	0.33–0.59	<0.001		7
*P* (log-rank test)		0.004					
							
*Diabetes, No/yes*						0.352	
No (*N* 1311/1453)	8.6 (7.1–10.4)	18.5 (16.1–21.2)	0.47	0.37–0.59	<0.001		10^b^
Yes (*N* 187/157)	15.6 (10.4–23.5)	44.0 (32.4–59.7)	0.35	0.21–0.59	<0.001		4
*P* (log-rank test)		<0.001					
							
*IFG, No/yes*						0.700	
No (*N* 1091/1235)	8.1 (6.5–10.0)	17.9 (15.4–20.8)	0.45	0.35–0.59	<0.001		10
Yes (*N* 220/218)	12.9 (7.6–17.9)	21.8 (15.6–30.3)	0.54	0.31–0.93	0.026		10
*P* (log-rank test)		0.334					
							
*Serum insulin, mU* l^−1^						0.059	
⩽16.0 (*N* 634/922)	5.7 (4.0–8.0)	16.5 (13.7–19.7)	0.35	0.23–0.51	<0.001		9
>16.0 (*N* 859/686)	12.1 (9.9–14.9)	26.1 (22.0–31.0)	0.47	0.36–0.61	<0.001		7
*P* (log-rank test)		<0.001					
							
*Triglycerides, mg* *dl*^−1^						0.536	
⩽154.0 (*N* 664/892)	8.4 (6.4–11.0)	15.7 (13.0–19.0)	0.54	0.39–0.75	<0.001		14^b^
>154.0 (*N* 831/717)	10.2 (8.2–12.9)	26.5 (22.4–31.2)	0.39	0.29–0.51	<0.001		6
*P* (log-rank test)		<0.001					
							
*Total cholesterol, mg* *dl*^−1^						0.623	
⩽217.8 (*N* 655/901)	10.3 (8.0–13.2)	19.7 (16.6–23.4)	0.52	0.38–0.71	<0.001		8.3
>217.8 (*N* 840/708)	8.7 (6.9–11.1)	21.4 (17.8–25.6)	0.41	0.30–0.55	<0.001		7.2
*P* (log-rank test)		0.469					
							
*HDL cholesterol, mg* *dl*^−1^						0.303	
⩽51.5 (*N* 725/785)	12.2 (9.9–15.2)	24.1 (20.4–28.3)	0.52	0.39–0.68	<0.001		9
>51.5 (*N* 716/793)	6.5 (4.7–8.8)	15.6 (12.7–19.2)	0.41	0.28–0.60	<0.001		11
*P* (log-rank test)		0.001					
							
*Serum creatinine, mg* *dl*^−1^						0.462	
⩽0.78 (*N* 758/796)	7.7 (5.9–10.1)	20.5 (17.2–24.5)	0.38	0.27–0.52	<0.001		8
>0.78 (*N* 737/813)	11.2 (8.9–14.1)	20.3 (17.0–24.3)	0.55	0.41–0.74	<0.001		11
*P* (log-rank test)		0.924					
							
*UAE, mg per 24 h*						0.208	
⩽9.3 (*N* 707/847)	4.3 (3.0–6.2)	9.2 (7.1–11.8)	0.48	0.30–0.74	0.001		21^b^
>9.3 (*N* 791/763)	14.1 (11.6–17.2)	33.8 (29.3–39.0)	0.41	0.32–0.53	<0.001		5
*P* (log-rank test)		<0.001					

Abbreviations:

BMI, body mass index; CI, confidence interval; HDL, high-density lipoprotein; HR, hazard ratio; IFG, impaired fasting glucose; N, number; NNT, number needed to treat; UAE, urinary albumin excretion.

IFG groups were defined after excluding individuals with diabetes.

Dichotomous variables could have one of the two values (men/women, etc). Interaction is adjusted for age and sex.

*P* (log-rank test): *P*-value for difference between incidence rates in high-risk vs low-risk groups in the control group.

To convert the values for glucose to millimoles per liter, multiply by 0.05551. To convert the values for insulin to picomoles per liter, multiply by 6.945. To convert the values for cholesterol to millimoles per liter, multiply by 0.02586. To convert the values for creatinine to micromoles per liter, multiply by 88.4. To convert the values for triglycerides to millimoles per liter, multiply by 0.01129.

A: Incidence of albuminuria in high-risk and low-risk subgroups. For continuous variables, subgrouping is based on median baseline values. B: Risk factor-treatment interactions for albuminuria in subgroups. C: Number needed to treat to prevent the development of albuminuria in one patient at 10 years.

a For each continuous variable, the test of interaction was calculated using the original continuous variable.

b Statistically significant difference in NNT between the subgroups.

## Notes

Lena Carlsson reports receiving consulting fees from AstraZeneca and owning stock in Sahltech; Lars Sjöström reports serving as a member of the board of Lenimen, receiving lecture fees from AstraZeneca and Johnson & Johnson and providing an expert statement on drug effects and weight-loss effects on obesity for AstraZeneca. The remaining authors declare no conflict of interest.
