# Supplementary material for: The incidence of albuminuria after bariatric surgery and usual care in swedish obese subjects (SOS): a prospective controlled intervention trial
Source: Int J Obes (Lond). 2014 Jun 10;39(1):169–75. doi: 10.1038/ijo.2014.72 (PMC4285618; doi:10.1038/ijo.2014.72)
Supplement: Supplementary Information [file ijo201472x1.doc]

**Online Supplement**

**The Incidence of Albuminuria After Bariatric Surgery and Usual Care in Swedish Obese Subjects (SOS): A Prospective Controlled Intervention Trial**

Lena M.S. Carlsson, M.D., Ph.D., Stefano Romeo, M.D., Ph.D., Peter Jacobson, M.D., Ph.D., Maria Antonella Burza, M.D., Cristina Maglio, M.D., Kajsa Sjöholm, Ph.D., Per-Arne Svensson, Ph.D., Börje Haraldsson, M.D., Ph.D., Markku Peltonen, Ph.D., Lars Sjöström*, M.D., Ph. D.

**Supplementary Figure 1. Mean body weight changes (%) and 95% confidence intervals (bars) over 15 years in the control and surgery groups of the SOS study according to the method of bariatric surgery.**

**
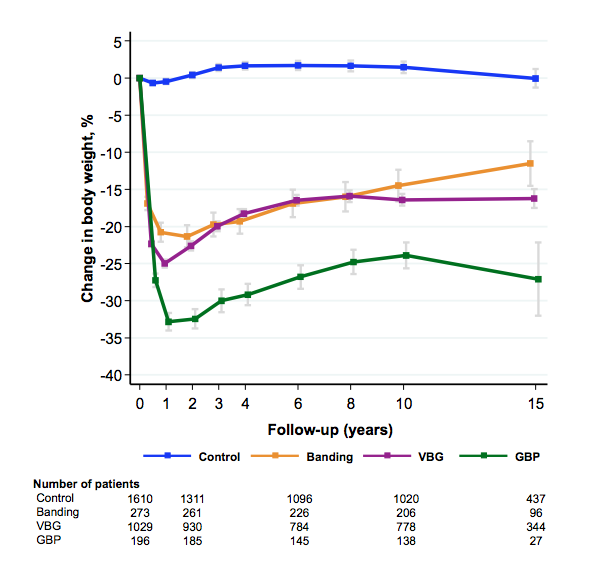
**
